# Brain Health Promotion with Métis People in Alberta: a qualitative study

**DOI:** 10.1002/alz70860_098932

**Published:** 2025-12-23

**Authors:** Pamela Roach, Shanaya Fischer, Meagan Ody, Lisa Zaretsky, Jennifer D Walker

**Affiliations:** ^1^ University of Calgary, Calgary, AB, Canada; ^2^ McMaster University, Hamilton, ON, Canada

## Abstract

**Background:**

The quality of culturally appropriate brain health promotion materials Indigenous people in Canada is lacking. Canada's National Dementia Strategy calls for additional work to improve brain health promotion and dementia prevention for Indigenous people as a priority. Health promotion and healthy brain aging for Indigenous communities also needs to be understood in a distinctions‐based and culturally specific way. Providing brain health promotion materials that are strengths‐based, while also being grounded in Indigenous knowledge systems and experiences is an urgent priority in Canadian health systems and plays an important part in enhancing patient safety and closing gaps in health inequities. The primary outcome of this project was to develop understanding regarding how the Brain Health Pro can be adapted to a Métis context in the province of Alberta, Canada.

**Method:**

Our overarching ethical approach embeds integrated knowledge translation into the development and implementation of brain health promotion material so that the research outcomes are directly applicable and useful to communities. Interviews were completed using the Métis visiting methodology (Keeoukaywin) to allow for the emergence of new themes. Data was co‐analysed using Indigenous approaches to thematic qualitative analysis.

**Result:**

Twenty Métis people in Alberta participated in a total of 63 interviews between July and November 2024. Participants felt that the current content and mode of delivery of Brain Health Pro was not appropriate for Métis people living in Alberta. Themes included a western dominance of brain health (including a lack of Indigenous people in the video content, no Métis Ways of Knowing; a lack of non‐virtual options (for those who may not have reliable internet access); and a need for holistic approaches to risk reduction (e.g. berry picking could provide physical activity, social interaction and healthy traditional food options and be an avenue for intergenerational knowledge mobilization).

**Conclusion:**

The work will be developed to facilitate spread, scale and adaption to other local Indigenous contexts. Additionally, the principles and methodology may be applied internationally to develop locally driven and culturally specific dementia care intervention for Indigenous populations or other underrepresented populations.

